# The identification *Mycobacterium tuberculosis* genes that modulate long term survival in the presence of rifampicin and streptomycin

**DOI:** 10.1038/s41598-025-04038-9

**Published:** 2025-07-01

**Authors:** Johana E. Hernández Toloza, Ye Xu, Tom A. Mendum, Bianca Sica Siedler, Rosalyn Casey, Huihai Wu, Kerstin Williams, Suzanne Hingley-Wilson, Johnjoe McFadden

**Affiliations:** 1 Department of Microbial Sciences, School of Biosciences, Faculty of Health and Medical Sciences, University of Surrey, Guildford, UK; 2 Bioinformatics Facility, Faculty of Health and Medical Sciences, University of Surrey, Guildford, UK

**Keywords:** Mycobacterium tuberculosis, Transposon library, Tolerance, Persister, Cell wall, Methylcitrate cycle, Bacterial genomics, Bacteria, Pathogens, Bacteriology, Antibiotics

## Abstract

In 2023, *Mycobacterium tuberculosis* (Mtb) caused 10.6 million new tuberculosis cases and 1.3 million deaths. The WHO proscribed treatment is not always successful, even when strains were sensitive to the antibiotics.as clinical Mtb populations contain phenotypically tolerant subpopulations, termed persisters*.* Here a Mtb transposon library was challenged with rifampicin (RIF) and streptomycin (STM) under conditions designed to identify genes that modulate persister frequency. Mutants with reduced survival in RIF were predominantly in genes associated with membrane integrity *e.g*. arabinogalactan assembly genes *cpsA/lytR/Psr*, whilst for STM, reduced survival was associated with toxin/antitoxin genes. Some mutations enhanced survival. For RIF these included the methyl citrate cycle genes *prpC, prpD* and *prpR*, and the trkA-C K^+^ uptake system genes ceoB and *Rv2690*, and for STM, the resistance associated gene, *gidB*, and anion-transport genes *Rv3679c* and *Rv3680c*. Few genes overlapped the RIF and STM selections, demonstrating that survival mechanisms were antibiotic-specific. Directed deletions of Δ*prpD* and Δ*fadE5* confirmed their predicted enhanced and reduced RIF fitness respectively. The study identified genes that modulate not only persister frequency but also resistance and tolerance, and demonstrates that the mechanisms that produce these phenotypes are diverse and antibiotic-specific.

## Introduction

In 2023, *Mycobacterium tuberculosis* (Mtb) was one of the leading causes of mortality from any infectious disease with 10.6 million cases tuberculosis (TB) and 1.3 million deaths^1^. Standard treatment requires a 4–6 month long multi-drug regimen. However, this extended treatment is not always successful and up to 15% of patients relapse, even when the infecting strains are fully drug susceptible. These relapses are thought, at least in part, to result from populations of bacteria that are in a phenotypic state that makes them refractory to the antibiotics, despite the strains being drug-sensitive.

The existence of such antibiotic-refractory bacteria was recognised at the very advent of the antibiotic era, when in 1944 Gladys Hobby recorded that around 1% of a streptococcal cell in culture survived long-term penicillin treatment^2^. These cells were named ‘persisters’ and were considered to be a phenotypically resistant sub-population induced by environmental stresses^2^. Subsequently, similar persister subpopulations were noted in other organisms, including *Pseudomonas aeruginosa* and *Escherichia coli*^3,4^. In Mtb populations, evidence for persisters has been identified not only *in vitro*^5^, but also in animal models^6–8^ and in humans^9–12^. A decade later in 1958, the related phenomenon of ‘tolerance’ was described by McDermott^13^ when he observed that anaerobiosis in ‘tubercule bacilli’ slowed the rate of killing by STM of the entire culture, not just a rare persister sub-population^14,15^.

This distinction between persister and tolerant populations is often characterised by their respective kill curve. Persisters have a biphasic kill curve, in which the bulk population of susceptible cells are rapidly killed leaving a minority population of persister cells that are killed more slowly. In contrast, the kill curve of tolerant populations have a slow, but constant kill rate, due to the reduced, but homogeneous sensitivity of the entire population^16^. Although closely related, there is evidence that the mechanisms that generate these two phenotypic states can be independent. For example growth arrest may induce tolerance in a population without altering the number of persisters, and indeed can mask the presence of any persister subpopulations^17^. These differences between persistence and tolerance can be subtle, and so to truly distinguish persisters from tolerant populations often single cell analysis such as microfluidic is required. In clinical situations there is evidence that *M. tuberculosis* populations are extremely heterogeneous, with populations of both persisters and tolerant cells, leading to multiple distinct killing rates^18^.

Persistence and tolerance are distinct from resistance in that they refer to a non-heritable phenotypic state in which cells are recalcitrant to antibiotics. Because the phenomenon is non-heritable, the progeny of persisters and of tolerant populations are antibiotic sensitive. This differs from resistant populations that have genetic changes that make the antibiotics less effective, and so the progeny inherit the antibiotic resistance profile of their parents. Classically, resistant bacteria can be distinguished from tolerant bacteria or persisters by having a raised minimum inhibitory concentration (MIC)^16^.

Although neither tolerant populations, nor persisters carry a genetically encoded resistance to antibiotics, genes have been identified that influence the frequency at which they arise. Some of the most intensively researched are toxin-antitoxin (TA) genes that have long been associated with increased dormancy and higher levels of both tolerance and persister formation^7,19,20^ in many organisms including Mtb^21,22^ and the related, model organism *M. smegmatis*^23–26^. However, the exact relationship between TA modules, dormancy, and recalcitrance to antibiotics is not straightforward. It is well recognised that slow growing or static cells can be less susceptible to antibiotics and that nutrition, stress, and growth stage can all be contributing factors^27^. However, these recalcitrant states do not arise as an indirect result of growth arrest but are rather a distinct, reversible, and programmed physiological state^21,25,28,29^. One of the first TA systems implicated in modulating antibiotic sensitivity was the RelA/SpoT system that has been shown to alter the frequency of persister formation by regulating a conserved stringent response. Mutants have altered levels of the regulatory metabolite (p)pGppp that cause changes in metabolite levels and in the cell wall that result in increased persister frequencies^30^. Other genes whose disruption has been identified as increasing antibiotic tolerance/persistence include genes such as those of the mycobactin exporting ESX-3 transport system that, under iron limited conditions, promote persister formation^31^ and the regulator, *mce3R*, that is involved in lipid metabolism and resistance to oxidative stress^32^. Mutations that sensitise Mtb to antibiotics have also been discovered, such as *phoY*, part of the phosphate regulating system. Mutations in *phoY* generate fewer persisters when phosphate starved or when in stationary phase^33^.

In an attempt to catalogue these tolerance and persistence modulating genes several studies have adopted whole genome approaches. Torrey *et al*^10^ in 2016 conducted a whole genome screen of randomly mutagenized Mtb co-challenged with both rifampicin (RIF) and streptomycin (STM) and identified non-synonymous SNPS in 36 genes that had altered fitness. The genes identified encoded functions that included phthiocerol dimycocerosates (pDIM) synthesis, glycerol catabolism and the TCA cycle. Further work demonstrated that SNPs in some of these genes were present in isolates from a longitudinal study of patients with Mtb infections that were recalcitrant to treatment, suggesting that antibiotic profiles of these mutants were clinically significant^10^. Using a similar approach, Xu *et al*^34^ exposed an Mtb transposon library to partially inhibitory concentrations of antibiotics, including RIF, to look for mutants with altered levels of survival. They identified 74 mutants with increased susceptibility to RIF including the *lytR*/*cpsA* cell wall group of genes, and genes associated with phosphate transport. Most recently, Bellerose et al., 2020 screened a transposon library^35^ in rifampicin treated mice to identify RIF hypersusceptible mutants, that were predominately associated with cell wall components.

In this study, we exposed an Mtb transposon library to either RIF or STM over an extended period and at concentrations that were designed to enrich for persisters*.* RIF remains one of four antibiotics used in the standard 1st line mutli-drug therapy for TB. STM is no longer a 1st line drug, but can still be used to treat patients with RIF resistant, or multi-drug resistant isolates^36^. By using two antibiotics with different mechanisms of actions we were able to compare the results and determine if the genetic requirements for persistence/tolerance were shared between antibiotics, or whether they were independent. Transposon library assays are particularly well suited to this type of study as they offer a robust and sensitive genome scale assay that can identify a wide range of mutants with altered fitness under specific selection criteria, such as an antibiotic challenge. When the transposon library is subjected to a selective pressure, mutants with altered fitness are enriched or depleted. The resultant changes in frequency of each insertion mutant in the library can then be determined by sequencing and the relative fitness of each mutant calculated^37,38^. Using these methods this study reports a genome wide assessment of the genes that modulate the frequency at which persisters, tolerance and resistance arise.

## Results

### *Mtb* H37Rv transposon library selection using RIF or STM

**Fig. 1:**
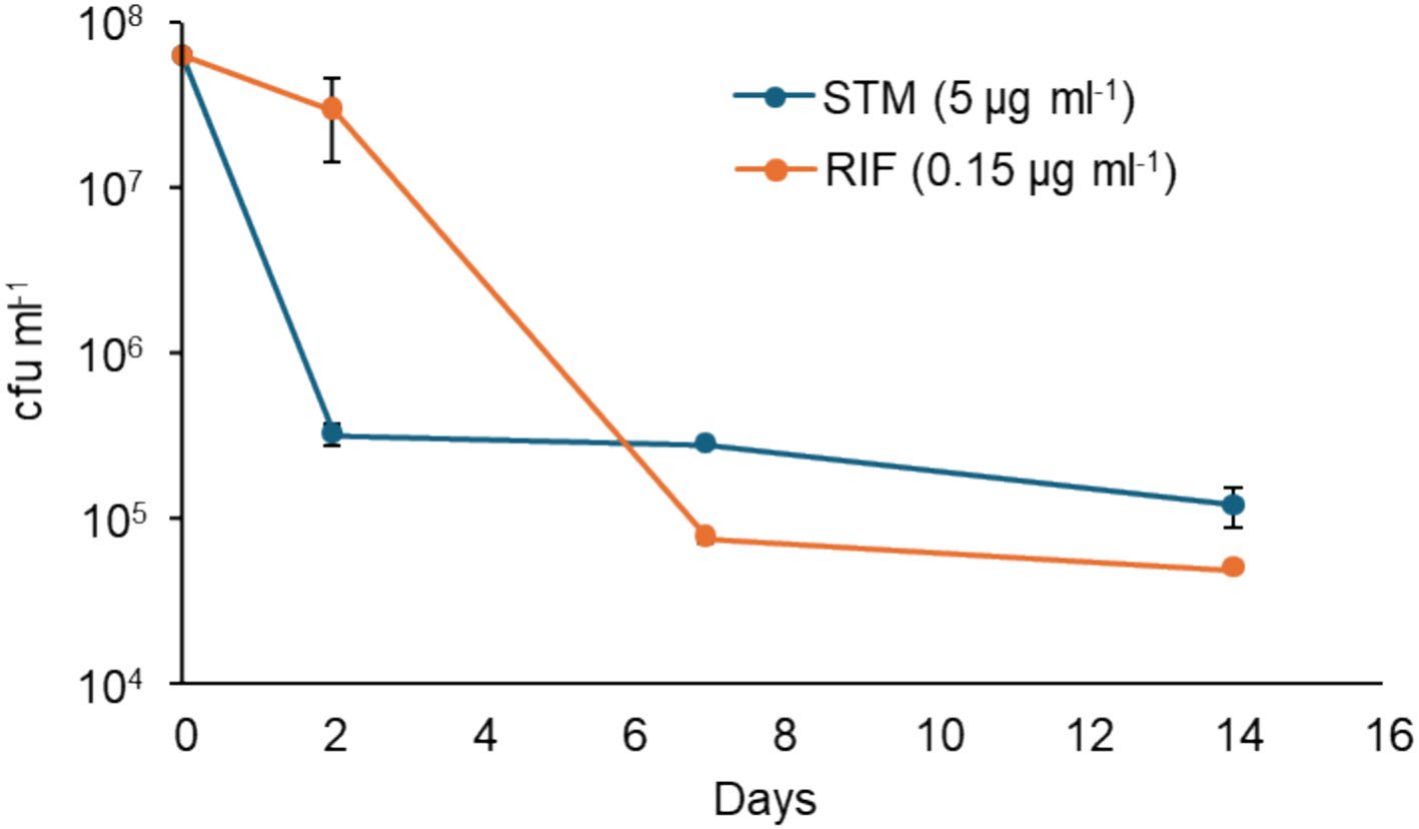
Kill curves for the transposon libraries. An exponential culture of the transposon library was exposed to 0.15 μg ml^-1^ (3 × MIC) of rifampicin, or 5 μg ml^-1^ (10 × MIC) of streptomycin. Results are representative of three biological replicates with the bars showing standard errors.

To design a transposon library selection to identify genes with altered persister frequency, we performed preliminary studies to determine antibiotic concentrations that generated the biphasic kill curve characteristic of persisters, *ie* had a rapid kill of the bulk of the population, and an antibiotic recalcitrant subpopulation (Supplementary Fig. 1), but still maintained an output library large enough to represent the libraries mutant diversity. This ensured that the concentration chosen balanced the competing requirements of ensuring the selection pressure was strong enough to differentiate between mutants with altered antibiotic fitness, whilst still maintaining a library diversity sufficient to allow identification of any changes in mutant abundance in subsequent analyses. The concentrations chosen were 3 times the MIC for RIF (0.15 µg ml^-1^) and 10 times the MIC for STM (5 µg ml^-1^). Both treatments rapidly reduced cell numbers by 2–3 orders of magnitude, after which numbers remained relatively constant throughout the rest of the experiment (Fig. 1).

Although designed to select for persisters, the selection also enriched for tolerant and resistant organisms. To help distinguish persister and tolerance promoting mutants, from resistant mutants, and so inform later interpretation of the data, we determined the number of heritably resistant bacilli throughout the antibiotic-killing experiment by sampling and enumerating the bacteria on plates containing RIF or STM. Prior to antibiotic exposure, the frequency of RIF and STM resistant mutants were 8 × 10^–5^ and 1 × 10^–4^ mutants CFU^-1^ respectively. These frequencies increased during the experiment, such that by day 14, 16% (RIF) and 25% (STM) of survivors were resistant mutants. However many of these resistant mutants, are likely due to spontaneous SNPs in genes such *rpoB* for RIF^39^ and *rpsL* and *rrs* for STM^40^. Because these SNPs are not associated with any specific transposon insertion, they will not be identified by the transposon library analysis so long as the library contains enough diversity that the SNPs occur in a wide variety of transposon mutant backgrounds so minimising any stochastic selection for a given transposon insertion site.

### Identification of genes affecting long-term survival frequency to RIF and STM

The Mtb transposon library contained 5 × 10^5^ individual mutants. Analysis of the transposon insertion loci predicted that this represented a saturation level of 67% of the TAs in the genome (50,228 sites from a total of 74,602 TA sites). Approximately 17,150 of these TA loci are deemed essential, whilst 6,659 have non-permissive motifs that are less likely to have transposon inserts^41^. Excluding these loci, leaves 60,040 TAs that are readily available for mutagenesis, giving a library coverage of 84% of the available insertion loci. In the antibiotic-treated output samples 45,254 sites (75%) and 34,409 sites (57%) were present in the library RIF and STM selected libraries respectively. Such levels of coverage have been previously shown to be sufficient to reliably identify genes whose frequency is modified by the selective pressure applied to the libraries^42,43^.

**Fig. 2:**
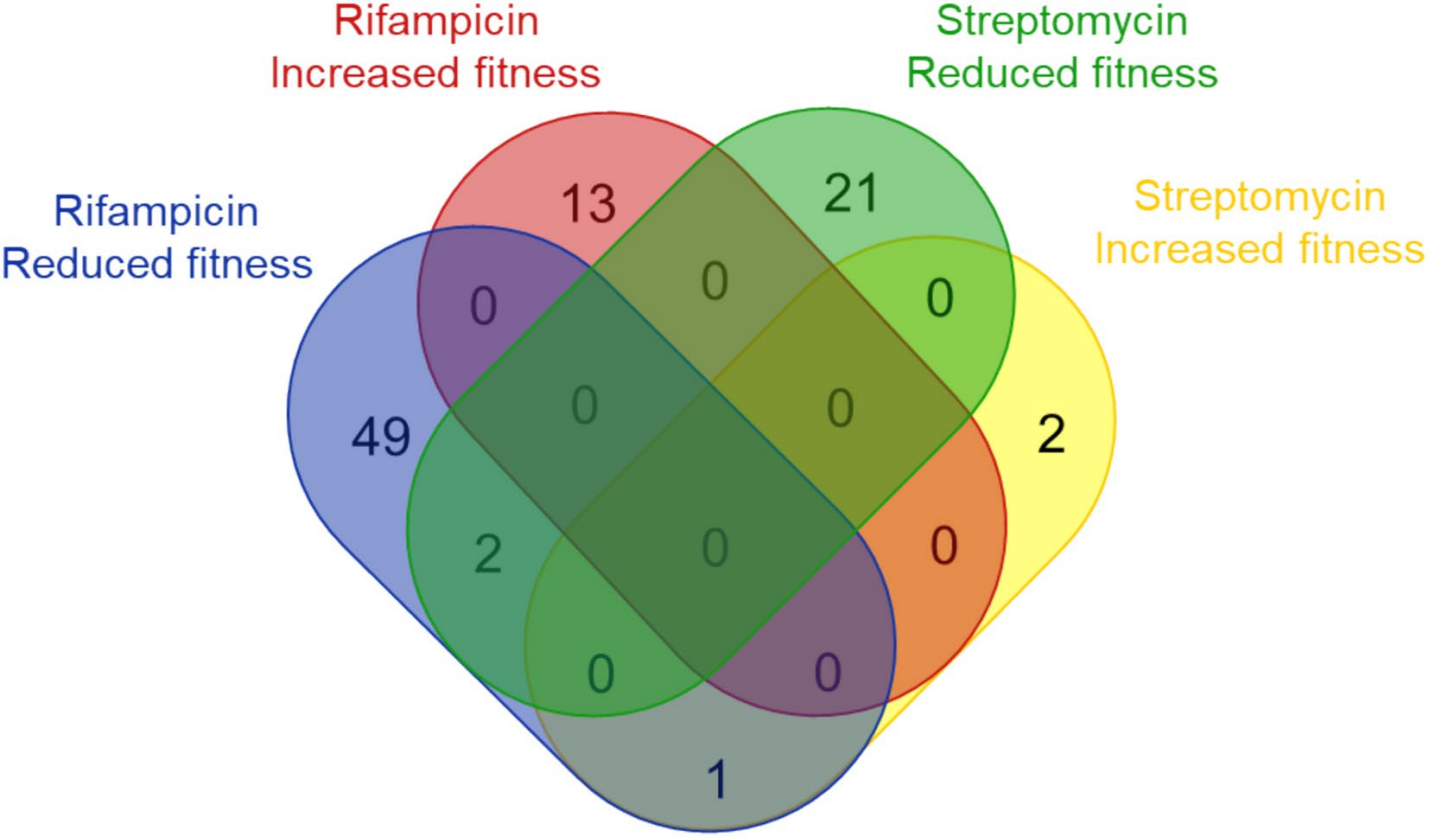
The relationship between mutants identified after RIF and STM treatment. A Venn diagram comparing the identified mutants (fold change of > 2 or < -2, and a q value of < 0.05) after antibiotic treatment with RIF or STM. Created by Venny 2.1^45^.

**Fig. 3:**
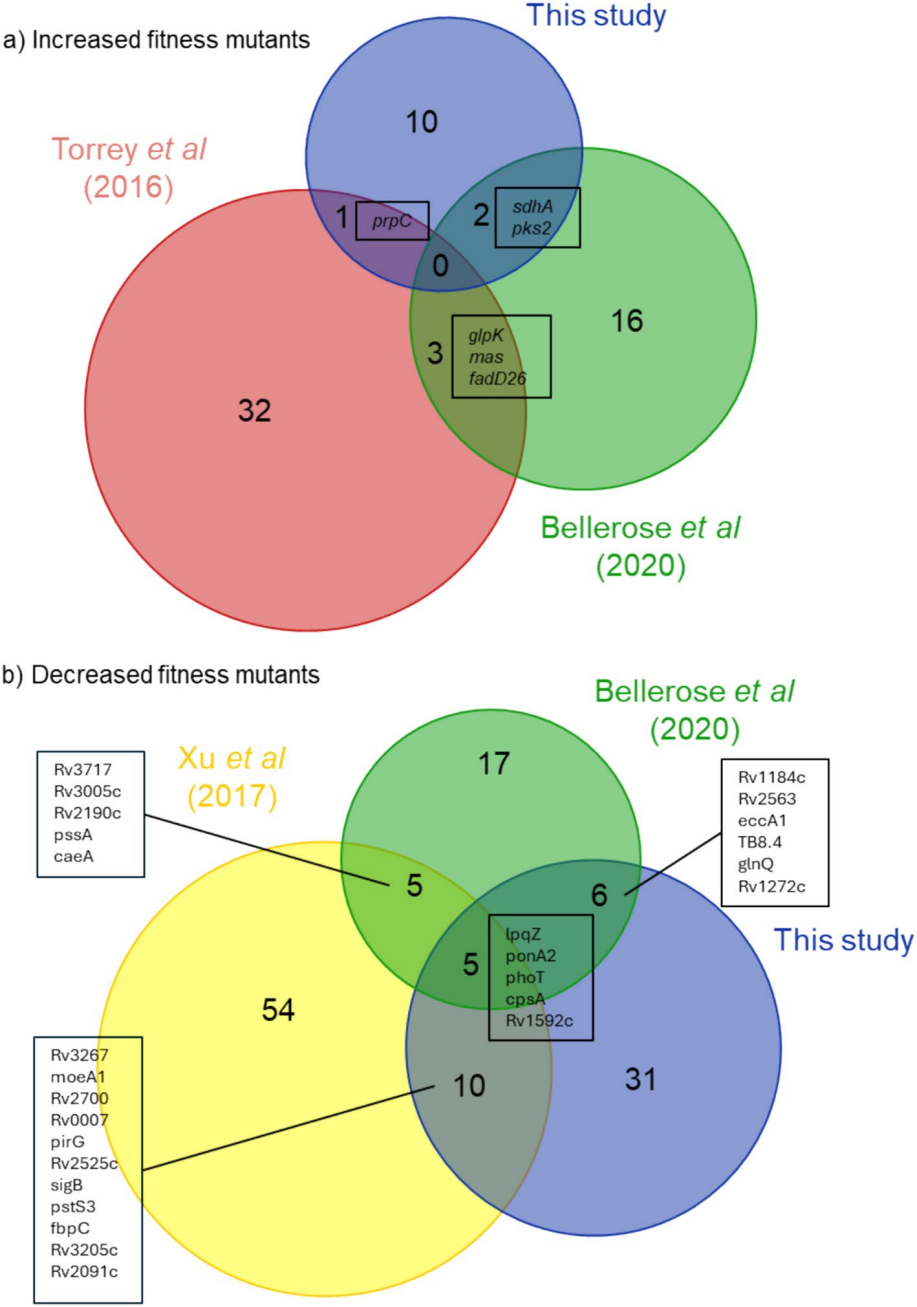
The relationship between mutants identified after RIF treatment in this study and other whole genome screens. Euler diagrams comparing mutants identified after treatment with RIF in published whole genome screens. Xu *et al*^34^ selected a transposon library with sub-lethal concentrations RIF (4 ng ml^-1^). Bellerose *et al*^35^ selected a transposon library with an inhibitory dose in a mouse model. Torrey *et al*^11^ selected a mutagenized library with inhibitory concentrations of both STM (10 µg ml^-1^) and RIF (1 µg ml^-1^) combined to identify mutants with increased fitness. Only *gidB* was common amongst mutants with increased fitness in the STM selection and Torrey *et al*^10^ (not shown). All overlaps are significant, chi squared, p < 0.001, except for the STM mutants of Torrey *et al*^10^ and those of this study were p = 0.002.

The transposon data was analysed with TRANSIT-2’s^44^ resampling method and mutants with both enhanced and reduced fitness identified. Using cutoffs of q < 0.05 and fold changes of <  > 2, a total of 52 and 23 reduced fitness mutants, and 13 and three (*gidB and* the anion transporter genes*, Rv3679c and Rv3680c)* enhanced fitness mutants were identified after RIF or STM treatment respectively (Fig. 2, Supplementary Data File 1). These genes strongly correlated with genes identified in previous genome scale studies that challenged mutant libraries with RIF and STM^10,34,35^ (Fig. 3, all with Χ^2^ p < 0.01).

All the mutants identified as having enhanced fitness after RIF treatment were distinct from those identified after STM (Fig. 2). For mutants with reduced fitness, only two were common to RIF and STM treatment, Rv2700, a hypothetical protein associated with the cell wall, and Rv0406c (a beta-lactamase) (Fig. 2), whilst one gene, *Rv3680*, part of anion transport mechanism, was identified as having enhanced fitness with STM and reduced fitness with RIF.

**Fig. 4:**
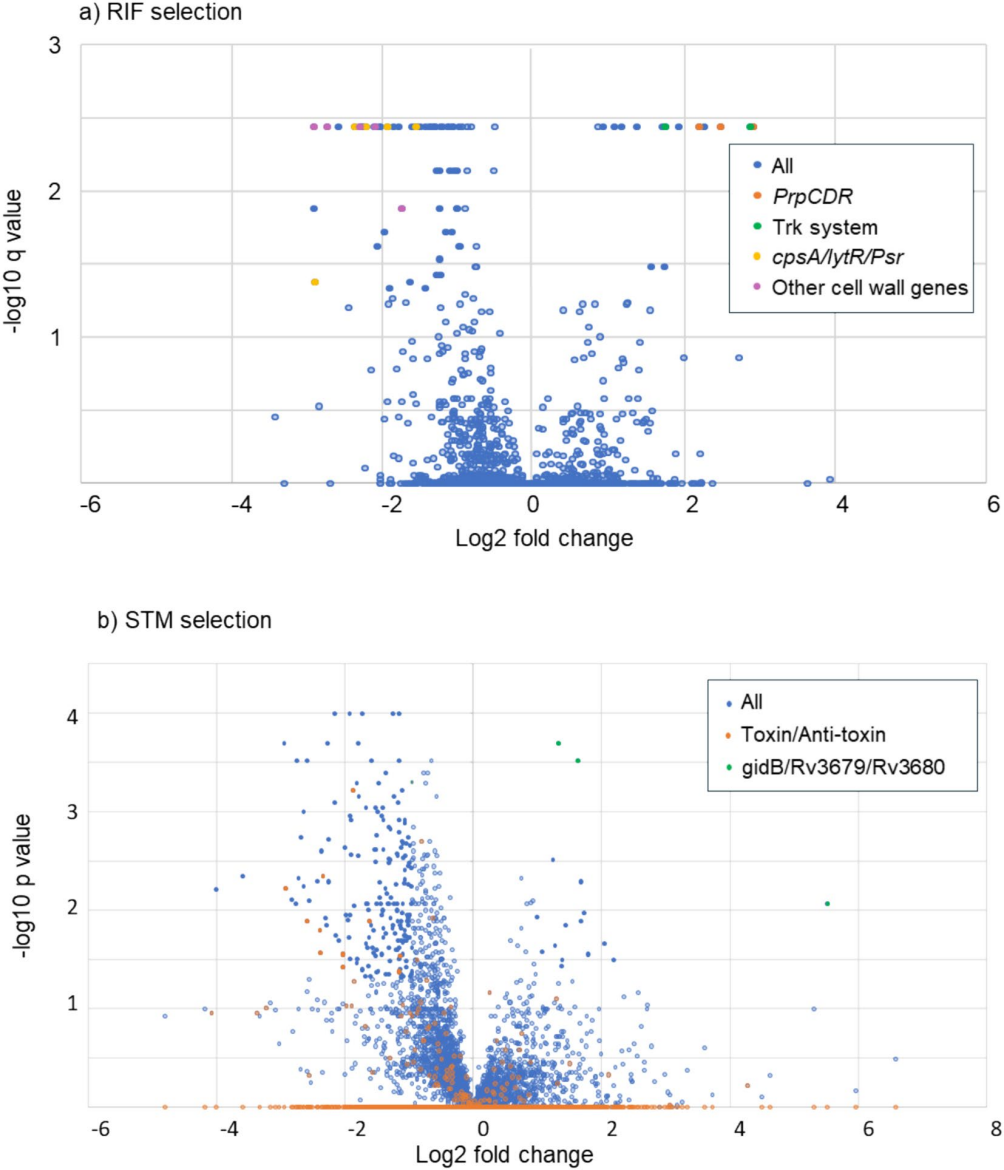
A volcano plot of (**a**) Rifampicin and (**b**) Streptomycin selected mutants. Each point represents an individual mutant and its predicted fold change phenotype and a probability. Mutants with fold changes of < 2, or > -2, and q > 0.05 (for RIF) or p > 0.05 (for STM) are faded.

**Fig. 5:**
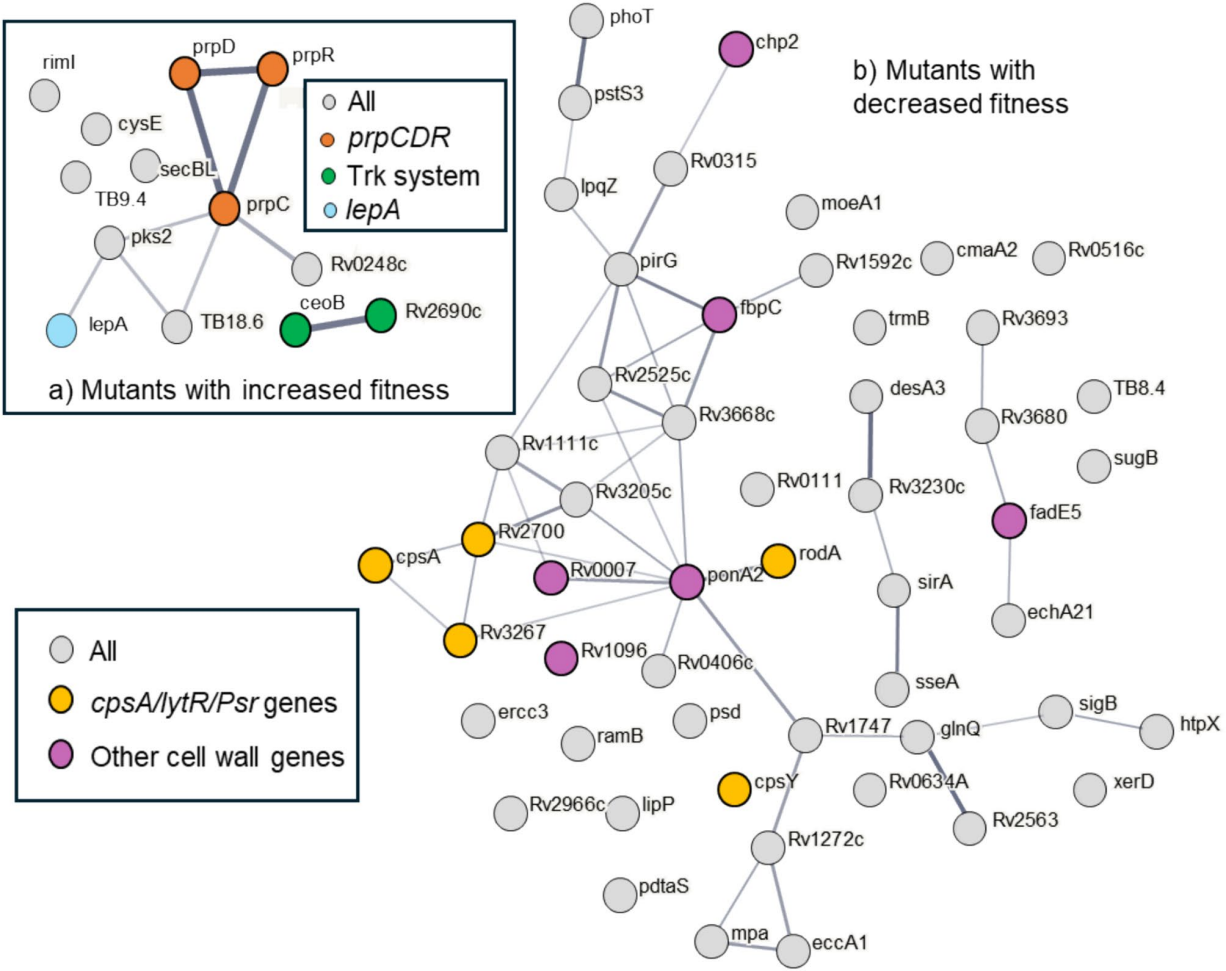
A gene association plot of mutants with (**a**) increased, and (**b**) decreased fitness when challenged with Rifampicin. Genes with q < 0.05 and fold change of more than 2 were submitted to STRING v12^83^. The thickness of the lines between plots indicate the confidence of the gene association.

**Fig. 6:**
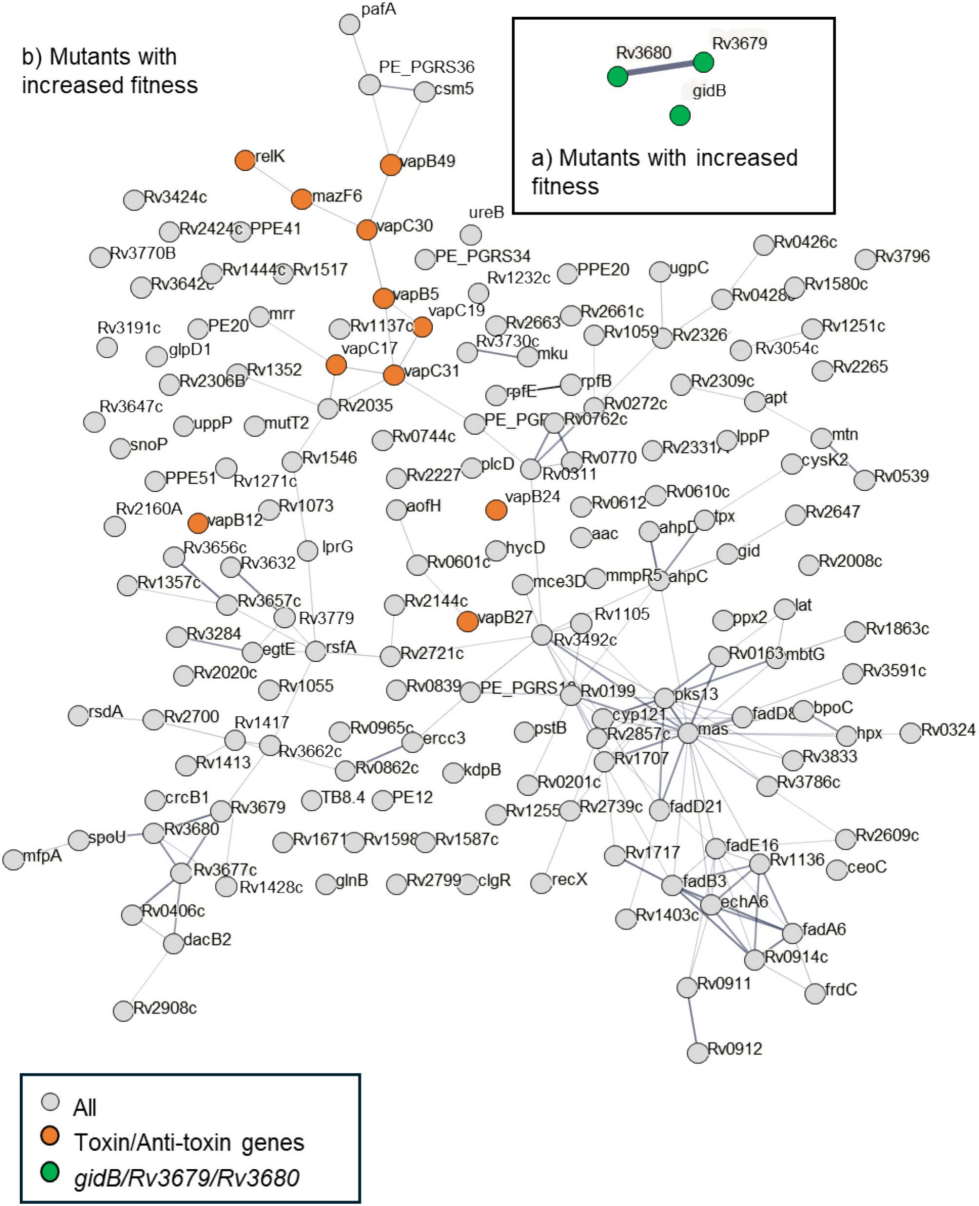
A gene association plot of significant mutants with (**a**) increased, and (**b**) decreased fitness when challenged with Streptomycin. Genes with *p* < 0.05 and fold change of more than 2 were submitted to STRING v12^83^. The thickness of the lines between plots indicates the confidence of the gene association.

Thirteen mutants were identified as having enhanced fitness with RIF (Figs. 4, 5 and Supplementary Data File 1). These included all three of Mtb’s dedicated methyl citrate genes, *prpCD* and their regulator *prpR*, (*p* < 0.001); two genes of the Trk K^+^ transport system, *ceoB* and the adjacent gene *Rv2690*, (*p* = 0.025); and *lepA*, a mutation known to induce rifampicin tolerance in *M. smegmatis*^46^. Three mutants had enhanced fitness with STM (Fig. 4, 6): *gidB,* which is known to promote resistance to STM^47,48^ and the operonic genes *Rv3679* and *Rv3680,* that are predicted to be part of an anion transport complex with unknown substrates but have been associated with nitrous oxide tolerance.

### Mutants with lowered fitness

Mutants with lowered fitness after antibiotic challenge were more numerous (Fig. 4–6, Supplementary Data File 1). Among the 52 mutants identified with RIF were five genes of the *cpsA*/*lytR*/*Psr* group, *cpsA*, *cpsY*, *rodA*, *Rv2700* and *Rv3267*^49^ (*p* < 0.001), that encode proteins that anchor arabinogalactan to peptidoglycan, along with a further 6 cell envelope associated genes: *chp2* (PAT synthesis^50^), *ponA2* (peptidoglycan crosslinking), *Rv0007* (peptidoglycan synthesis^51^), *Rv1096* (peptidoglycan deacetylase^51^), *fbpC* (trehalose dimycolate synthesis) and *fadE5* (lipid beta degradation). Two of these mutants, *ponA2* and *Rv0007*, are thought to interact, with *Rv0007* being required for *ponA2* function^51^, both were previously identified by Xu *et al*^34^ as being lost in transposon libraries subject to sub-lethal concentrations of RIF. STM treatment identified 23 mutants with lowered fitness, although no functional groupings were apparent (Supplementary Fig. 2). When the cutoff criteria were lowered, and *p* values rather than *q* values used, we identify 172 mutants with reduced fitness (Fig. 5–6), and noted a preponderance of toxin/anti-toxin mutants, *vapB5, B12, B19, B24, B27, B49, C17*, *C19*, *C30, C31*, *mazF6, and relK (Χ*^2^* p* = 0.015).

### *ΔfadE5* mutants have altered fitness.

**Fig. 7:**
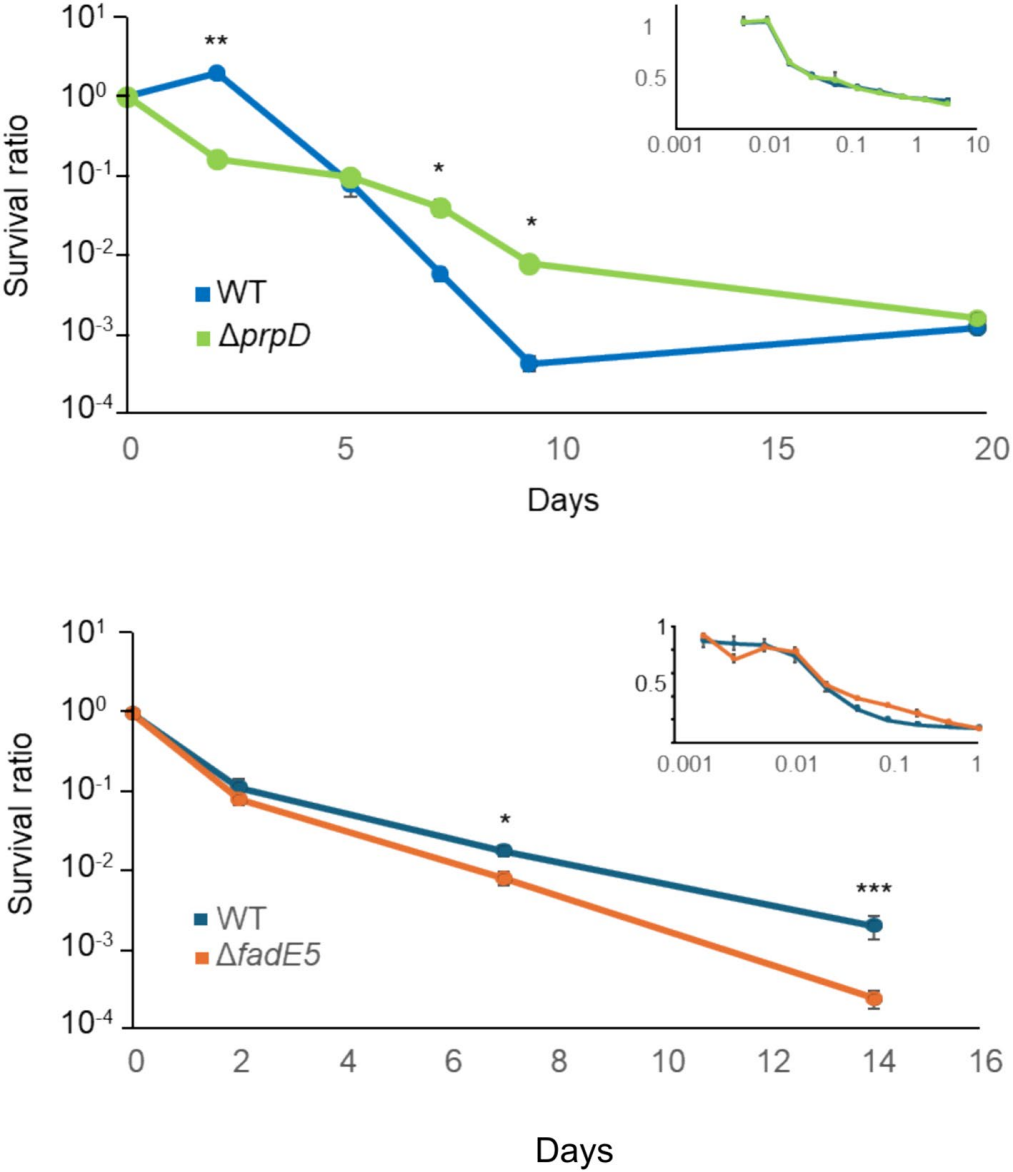
Kill curves of *∆prpD and* Δ*fadE5* challenged with RIF. Kill curves of (**a**) *∆prpD (∆Rv1130)* and (**b**) Δ*fadE5* (∆*Rv0244*) mutants treated with 1 µg ml^-1^ rifampicin compared with wild-type *Mtb*. Values are mean ± SEM of at least three biological repeats. Statistical differences were calculated using unpaired t-tests. *p < 0.05, **p < 0.01, ***p < 0.001. Insets are the MIC for the mutants (from Supplementary Fig. 3), of RIF concentration (µg ml^-1^) plotted against the ratio of the treated culture and the no antibiotic control.

To confirm our transposon-based predictions and to further investigate the antibiotic phenotypes of the mutants, we generated targeted deletions of two RIF selected mutants: *prpD* (this knockout included the N terminal region of *prpC,* in much the same way that a transposon *prpD* insertion likely disrupts the operonic expression of *prpC*), which was identified as a mutant with increased fitness, and fadE5, which was identified as a mutant with lowered fitness. Time-kill experiments were performed to determine their persistence/tolerance phenotypes, and MICs carried out to measure any resistance phenotype. Both *∆prpD* (predicted fold change of × 4.9) and *∆fadE5* mutants (predicted fold change of × 0.22) behaved as expected when exposed to RIF, with *∆prpD* having significantly more survivors and *∆fadE5* producing significantly less (Fig. 7). The dynamics of the kill curves were both indicative of changes in tolerance rather than persisters, but definitive conclusions are difficult to draw without single cell analysis using microfluidics, which would constitute future works. Importantly no change in the MIC was observed (Fig. 7, Supplementary Fig. 3), demonstrating that these mutations did not infer a resistance phenotype. These results confirm that the transposon library screen identified genes that both increase and decrease the fitness of Mtb in ways that is not attributable to heritable changes in resistance.

## Discussion

Transposon libraries are a comprehensive and sensitive tool to generate genome-scale inventories of mutants that alter fitness under a given environmental condition^34,35,43^. For the library to be effective they must contain sufficient diversity, both before and after selection, to ensure good coverage of the genome and allow statistically significant differences in gene frequencies to be detected^37^. In these experiments we had to balance the need to select for the rare persister subpopulations that we hoped to analyse, whilst simultaneously retaining sufficient diversity to ensure that the library remained representative. The library used in this study contained 5 × 10^5^ mutants and our experimental inoculum was approximately 100 times this number. Antibiotic selection reduced the population by ~ 1000-fold; allowing us to recover approximately 10^6^ cells that was sufficient to provide a statistically valid sampling of the Mtb genome of ~ 4,000 genes and minimise any stochastic gene selection.

Both RIF and STM treatment of the transposon library changed the relative abundance of a wide variety of mutants, that were associated with long-term survival in the presence of the antibiotic. The genes identified correlated strongly with previous studies^10,34,35^, with many, but not all, of the genes identified in this work being common between studies. This both validates our data and implies that the genes uniquely identified in our study are also associated with antibiotic fitness.

Although the timing of the selection, and the antibiotic concentrations, were optimised to enrich for persisters we did inevitably also identify genes associated with resistance and tolerance. This is because it is difficult, if not impossible, when working with bulk populations to design out co-selection of resistance and tolerance associated mutants alongside any persister enhancing mutants. We did observe a strong selection for resistant organisms in our experiments, but despite this, we did not identify many transposon-associated resistance mutants in our analysis. This is because most of the resistant bacilli that were observed are likely due to SNP-mediated spontaneous resistance^39,40^ and so did not lead to the enrichment of any individual transposon insertion locus and as such they are not identified in the analysis.

As well as resistance associated mutants, we also identified many mutants associated with tolerance. In the context of our transposon library assays such co-selection is almost inevitable as the very distinction between tolerance and persister modulating mutants is challenged. Our transposon library is a mix of ~ 50,000 unique mutants, making any single mutant, including tolerance associated mutants, inherently rare, even after the selective pressure has been applied. This makes the behaviour of any single mutant in the tranposon library indistinguishable from that of persisters, which are defined as being a rare tolerant subpopulation. In similar experiments, when Torrey et al., 2016^10^ subjected a mutagenized population of Mtb to a combination of RIF and STM they also found a mix of tolerance and persister-enhancing, as well as some genetically resistant mutants, including the STM resistance mutant *gidB*. Only when individual mutants are created and assayed can an attempt be made to distinguish tolerance from persistence, and even then, such distinctions can be difficult to determine without single cell analysis.

### The mycobacterial cell wall—the front-line defence against RIF

Our RIF selection identified 52 mutants with reduced fitness, a significant number of which were in cell wall associated genes. Such mutants are of particularly interest as any gene target that synergises with RIF, could form the basis of novel drugs with the potential to enhance the effectiveness of RIF and so reduce rates of treatment relapse. Several of these cell wall mutants, *cpsA2*, *cpsA*, *cpsY, rodA*, *Rv3267c *and *Rv2700* were in genes that are members of the LytR-Cps2A-Psr family involved in arabinogalactan transfer and attachment to peptidoglycan^52–54^. Of these genes, *Rv2700* and *Rv3267c* deletion mutants have been reported to have much reduced IC50s/MICs for RIF^53^, indicating that these mutants exhibit reduced RIF resistance, rather than persistence or tolerance. These changes in resistance were antibiotic specific, as the same mutants did not have enhanced sensitivity to either isoniazid or ethambutol^53,54^, whilst in our STM screen these mutants were not selected. We identified a further six mutants in genes involved cell wall synthesis, *chp2*, *fbpC, fadE5, Rv1096* and the functional pair, *ponA2 and Rv0007*^51^. *Rv0007* mutants have previously been identified as having a reduced ability to survive inhibitory concentrations of RIF^34,55^, but importantly, its MIC is reported to be unchanged^34,56^, confirming that knockouts of this gene are indeed low persister/tolerance mutants. Whilst *fadE5* was one of the two knockouts used to validate our selection, and was shown to have a tolerance phenotype with reduced kill rates but no change in MIC.

The mycobacterial cell wall is normally an important barrier to host-effector mechanisms and antibiotics. By disrupting the cell wall’s structures these mutants likely increase cell wall permeability, allowing more rifampicin to enter the cell, so increasing sensitivity to RIF^35,57^. Although these increases in cell wall permeability seems to affect RIF, the mutants do not seem to affect sensitivity to STM, as none of these genes were found in our STM screen, supporting our conclusions, and that of other studies^34^, that the cell wall’s protective properties are especially relevant to challenge with RIF.

### Antitoxin-toxin mutants promote mycobacterial long term survival in STM

Our initial STM selection analysis identified 23 mutants with reduced fitness, but with no obvious functional enrichment. If we relaxed the criteria to use *p* value < 0.05, rather than the *q* value, we found that toxin/antitoxin(TA) genes were significantly enriched, with 12 TA genes in the now 172 mutants. Toxin/anti-toxin genes are typically shorter than other genes, and so have less transposon insertion sites (a median of 3 TA loci compared to a 8 for all genes) and so need higher levels of transposon saturation to reach a significance threshold^58^, making there identification in a transposon screen inherently prone to type II error *ie* falsely reporting no change in abundance. Using p-values rather than a q-value compensates for this, but at the cost of increasing the risk of Type I errors, *ie* falsely reporting changed abundance.

Toxin:anti-toxin (TA) modules have been suggested as one of the mechanisms by which Mtb is able to enter the slow or non-growing states that are often associated with tolerance or persistence^59,60^. The Mtb H37Rv genome is estimated to contain 149 of these TA genes, constituting some 79 TA systems, that are thought to be regulated, and to function, independently of each other^61–63^, including in the presence of STM^64^. Two of the 12 TA mutants identified in our STM screen have been characterised. VapC30 has been shown to be associated with slow growth, dormancy and persistence^65,66^. In *M. smegmatis*, the VapC30 ortholog regulates glycerol metabolism via a specific ribonuclease activity to co-ordination the anabolic demands of the cell (*e.g.*, fast or slow growth) with glycerol catabolism and so ensuring energy-producing and energy-consuming reactions of the cell are in balance^65^. The other well characterised toxin identified as depleted in our screen was *mazF6* that, along with genes *mazF5* and *mazF1, a*re known to facilitate a state of reversible bacteriostasis that can enable survival upon antibiotic exposure^67–69^. The established roles of these two TA mutants in modulating bacteriostasis and in surviving antibiotic exposure, implies that despite utilising lowered statistical stringency, the mutants we identify were genuinely depleted in the STM selection.

### Disruption of the methyl citrate cycle promotes long term survival in RIF

Our RIF selection identified 13 mutants with enhanced survival, which included mutants in genes encoding all three of the dedicated enzymes of the methyl citrate cycle (MCC), *prpC*, *prpD*, and their regulator *prpR*, indicating that dysfunction of the MCC increases survival under antibiotic stress. The MCC is a heavily regulated pathway that in the presence of propionyl-coA uses PrpC, PrpD, a (methyl)isocitrate lyase and part of the tricarboxylic acid cycle to metabolise propionate to pyruvate via a series of potentially toxic intermediates. When this pathway is blocked, metabolism is disrupted either directly by the toxic intermediates, or indirectly due to collateral changes to metabolism^70^, that ultimately lead to growth being inhibited, or even prevented^71,72^.

A role for the MMC in altering RIF susceptibility has been previously observed by Hicks *et al*^11^ who identified SNPs in *prpR* and *prpC*, but not in *prpD,* as prevalent in drug-resistant clinical isolates. They further demonstrated that in vitro*,* a *prpR* knockout (which have severely reduced *prpDC* expression) was tolerant to RIF, but only in the presence of propionate^70,73^.

The identification of the three MCC mutants in our library screen and the properties of our directed *prpD* knockout, confirmed that, at least under our conditions, the MCC tolerance phenotype occurred even in the absence of propionate implying that the MCC remained active under such conditions. Metabolic models and ^13^C labelling experiments predict this to be possible, with the cycle running in reverse to produce propionyl CoA for lipid production^74,75^. This activity could then still produce the toxic intermediates and metabolic changes that cause the growth disrupting, and tolerance phenotypes^11,76^. The absence of a tolerance phenotype by Hicks *et al*^11^ when propionate was not present maybe simply be due to difference in the experimental detail with differing timelines (6 v 14 days) and RIF concentration (0.5 v 0.15 µg ml^-1^), particularly as in our data tolerance is only apparent after day 7.

The other discrepancy between our results and those of Hicks *et al*^11^ is that although *prpC* and *prpR* were identified in both studies*, prpD* was observed only in our transposon screen. Tolerance in transposon mediated Δ*prpD* has been observed in a separate study by Quinonez et al., this time to isoniazid^70^, but again only in the presence of propionate. It may be that the transposon insertion knockout strategy, used in our library and by Quinonez *et al*^70^, disrupts the operonic expression of the downstream *prpC* gene, and so mutants phenocopy the Δ*prpR* used by Hicks *et al*^11^, whilst the effect of *prpD* SNPs on *prpC* function in the clinical isolates screened by Hicks *et al*^11^is minimal.

### Potassium transport disruption promotes long term survival in RIF

The other notable gene grouping identified amongst mutants with enhanced survival in the RIF treated samples *was ceoB* and the adjacent *Rv2690c* gene. *CeoB* is a component of the Trk system, a K^+^ uptake system that is known to have major roles in osmotic stress tolerance, internal pH maintenance, regulation of protein activity and the control of bacterial virulence^77^. Exactly how disrupting this system leads to increased fitness under RIF stress is unclear but a lack of potassium can lead to non-replicating states in *Mtb,* which are commonly associated with persistence/tolerance mechanisms^78^.

## Summary

Our study identified Tn mutants that had both enhanced and reduced fitness in the presence of RIF and STR. Amongst these mutants were genes associated with both tolerance and persistence, as well as some genes associated with resistance. Surprisingly, very few genes were common to both RIF and STR treatments, implying that the mechanisms that result in persistence and tolerance phenotypes were specific to each antibiotic. Several of the genes identified have previously been associated with changed levels of fitness under antibiotic stress, strongly arguing for the effectiveness of our screen and the relevance of the additional novel genes identified here. We confirmed the fitness phenotypes of two of the novel mutations, Δ*prpD* and *ΔfadE5*. Mutations in some of genes identified have been observed in clinical studies^11,46,79^, demonstrating that the library screen did identify clinically relevant targets, and therefore is providing new potential targets for TB drug development. To conclude, our findings identify a wide range of genes, unique to each antibiotic, that are involved in both increasing or decreasing the fraction of persisters or tolerant cells in the population of Mtb.

## Methods

### Strains, media culture and laboratory conditions

*Mtb* was grown at 37 °C in Middlebrook 7H9 containing 0.2% glycerol and 0.05% Tween80® with shaking (200 rpm), or on Middlebrook 7H11 agar. Time-killing experiments used a modification of the method described by Langmead and Salzberg^80^. To optimise the antibiotic concentration for the library selction, antibiotics were added to exponential phase (OD_600_ 0.6–0.8) cultures at 3, 5 and 10 times the MIC: 0.09, 0.15 and 0.3 µg ml^-1^, respectively for RIF and 1.5, 2.5 and 5 µg ml^-1^, respectively for STM. Samples were enumerated by culture before antibiotic addition and after 2, 7 and 14 days. At least 3 biological and 3 technical replicates were carried out for each experiment.

### Selecting transposon library by RIF or STM exposure

A *himar*1 H37Rv transposon library of approximately 5 × 10^5^ individual mutants was generated as described by Mendum *et al*^43^ and frozen in aliquots at -70 °C. Triplicate aliquots were thawed and cultured until exponential phase (OD_600_ ~ 0.7). For each replicate, an initial sample was removed, and cultured on agar until confluent, the material was collected and frozen to give ‘input’ samples. The remaining cultures were split in two, and either RIF (0.15 µg ml^-1^) or STR (5 µg ml^-1^) added. Samples were enumerated on days 0, 7 and 14, on non-selective plates, to determine the overall number of surviving bacteria, and on selective plates to determine the number of resistant bacteria. At day 14 the cultures were harvested by centrifugation, washed in media, plated, incubated until confluent and stored at -70 °C to give ‘output’ samples.

### Library DNA extraction

DNA was extracted from each input and output sample. Samples were centrifuged, the cryopreservant removed, and the pellet resuspended in TE, pH 8.0 (Sigma). An equal volume of 2:1 methanol:chloroform (Sigma) was added and the solution rocked by hand for 5 min. The suspension was then centrifuged at 4000 rpm for 10 min and the aqueous and organic phases carefully removed from the bacterial mass which was dried for 2–3 h. TE buffer (5 ml) containing 100 µg ml^-1^ lysozyme (Sigma), 1% SDS and 100 µg ml^-1^ of proteinase K (Sigma) was added to the pellet and the mix incubated at 50 ºC for 3 h. The viscous solution was transferred into a clean tube containing an equal volume of phenol:chloroform:isoamyl alcohol 25:24:1 (Sigma) and mixed by hand for 15 min. The mix was centrifuged, and the aqueous phase removed to a new tube and extracted once again with an equal volume of chloroform. The aqueous phase was then transferred to a new tube, and the DNA precipitated with 0.1 volumes of 3 M sodium acetate (Sigma) and × 0.7 volume of isopropanol (Sigma). Recovered DNA was washed twice with 70% ethanol and dissolved in molecular grade water. DNA quality and quantity was measured on 0.8% agarose, and by Nanodrop (Thermo Scientific).

### Processing DNA for next generation sequencing

Approximately 5 μg of the DNA was suspended in 130 µl of molecular grade water. The suspension was transferred to a cuvette and the DNA sheared using a Covaris Sono 7 machine with settings of Incident Power—105, Duty Factor – 5% for 200 cycles/burst for 80 s. Resultant DNA fragments were quantified and checked on agarose to confirm shearing.

The DNA fragments were blunt ended using the NEBNext End Repair Module (New England BioLabs) by mixing 1–5 µg of DNA with 10 µl NEBNext End Repair buffer (10x), 5 μl NEBNext End Repair Enzyme mix and water to a total volume of 100 µl and incubated at 20 °C for 30 min. The product was cleaned with a QiaQuick PCR cleanup kit (Qiagen) and resuspended in 50 μl water. A 3’ A was added to the blunt-ended DNA, using the NEBNext dA-Tailing Module (New England BioLabs) following the manufacturer’s instruction: recovered DNA was mixed with 5 µl NEBNext dA-Tailing reaction buffer (10x), 3 μl Klenow fragments (3’-5’ exo) and molecular grade water to 50 μl, incubated at 37 °C for 30 min and cleaned with a QiaQuick PCR cleanup kit and eluted into 50 μl water.

An adapter with a 3’ ‘T’ overhang was generated by mixing 100 μM of oligonucleotide Adap1 and Adap2 (Supplementary Table 1) in 50 μM MgCl_2_, heating at 95 °C for 5 min, and then cooling slowly to room temperature. This adapter was ligated to the A-tailed DNA by adding 4 μl of the 50 μM adapter to 250 ng A-tailed DNA (100–200 molar excess) with T4 ligase (Promega) and incubating for 2 h at room temperature. The resultant product was cleaned up with the QiaQuick PCR cleanup kit (Qiagen) with 4 PE washes, and eluted into 50 μl molecular grade water.

Loci adjacent to the Mariner transposon were amplified with primers MarA to MarJ, and primer IS6 (Supplementary Table 1). Cycle conditions were 98 °C for 30 s, followed by cycles of 98 °C for 10 s, 58 °C for 10 s and then 72 °C for 30 s. Cycle numbers were optimised in 10 µl real-time reactions containing 5 μl Phusion High Fidelity PCR master mix with GC buffer (New England BioLabs), 0.5 μl × 20 EvaGreen (Biotium), 2 pmol ul^-1^ of each primer, and 1 μl of the DNA a to give maximal amplification but without entering the artefact inducing plateau phase of the reaction. Once the number of cycles was optimized, the PCR were repeated in larger, preparative volumes of 4 × 50 μl each. To remove PCR artefacts such as primer dimers and get the appropriate fragment sizes, the PCR products were separated on a 1% agarose gel and fragments of between ~ 400–600 bp excised and cleaned with the QiaQuick Gel extraction kit (Qiagen), using 6 PE washes. The final product was eluted into 50 μl molecular grade water after a 2 min incubation at RT and fragment size and concentrations determined on a BioAnalyser (Agilent 2100). Samples were Illumina sequenced, with double reads of at least 100 bp, and double indexing.

Sequencing results were returned as fastq files and processed:

1. fastqFilter.py : filters input read sequences where end nucleotides with a fastq score < '#' were removed.
2. transposonFilter.py : filtered out non-transposon reads, by removing anything that does not contain the transposon insert site of ‘TGTTA’ near the start of the read.
3. aligned to the *Mtb* H37Rv genome (NC-018143) using Bowtie2 ^81^.
4. count Tn PCR dedup.py : counts the number of inserts at each position with mapping quality <  = 30 and removes those with the same random Adap1 index and insertion loci, so removing artefactual PCR duplicates.
5. Gene essentiality analysis using the TRANSIT resampling method. We used a cut-off fold change ± twofold and a *q* value of 0.05, and visualised with STRING version 12^82^.

### RIF Time-Kill experiment with deletion mutants

The *prpD*(∆*Rv1130*) knockout was constructed by PCR amplifying homologous regions upstream and downstream of *prpD* (Supplementary Table 2) and inserting them into pYUB245 as described by Bardarov et al.^83^. Mycobacteriophage for the *fadE5* knockout were kindly provided by Prof. William Jacobs Junior of Albert Einstein College of Medicine. Both wild type and KO mutants were cultured to an OD_600_ 0.5 ~ 0.8. Rifampicin (Sigma) was added to the cultures to a final concentration of 1 μg ml^-1^ (33 × MIC) and incubated at 37 °C for 2–3 weeks. Aliquots were taken at time points and enumerated by plating.

### MIC assays of deletion mutants.

The wild-type *Mtb* H37Rv and KO mutants *prpD and fadE5* were cultured to an OD_600_ ~ 0.5, diluted 1:10 and inoculated into antibiotic dilution series arrayed in 96 well plates, and incubated at 37 °C for one week and the OD_600_ value of each well was measured using a microplate reader.

## Supplementary Information

Supplementary Information 1.

Supplementary Information 2.

## Data Availability

Sequence data for these works is available on the NCBI Sequence Read Archive accession number PRJNA1102339.
