# Supplementary material for: The identification Mycobacterium tuberculosis genes that modulate long term survival in the presence of rifampicin and streptomycin
Source: Sci Rep. 2025 Jul 1;15:21746. doi: 10.1038/s41598-025-04038-9 (PMC12216072; doi:10.1038/s41598-025-04038-9)
Supplement: Supplementary file 1 — Supplementary Information 1. [file 41598_2025_4038_MOESM1_ESM.docx]

Supplementary Data

Identification of a diverse range of rifampicin and streptomycin persistence-modifying genes in *Mycobacterium tuberculosis* by transposon mutagenesis.

Johana E. Hernandez Toloza, Ye Xu, Tom A Mendum, Bianca Sica Siedler, Rosalyn Casey, Huihai Wu, Kerstin Williams, Suzanne Hingley-Wilson and Johnjoe McFadden


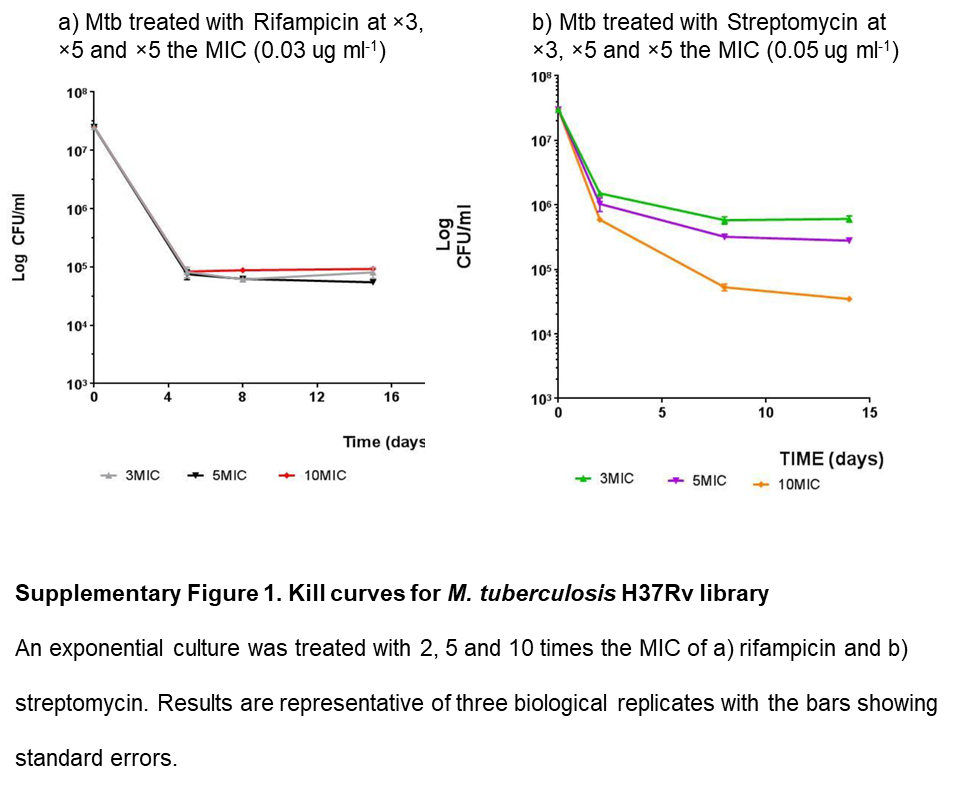


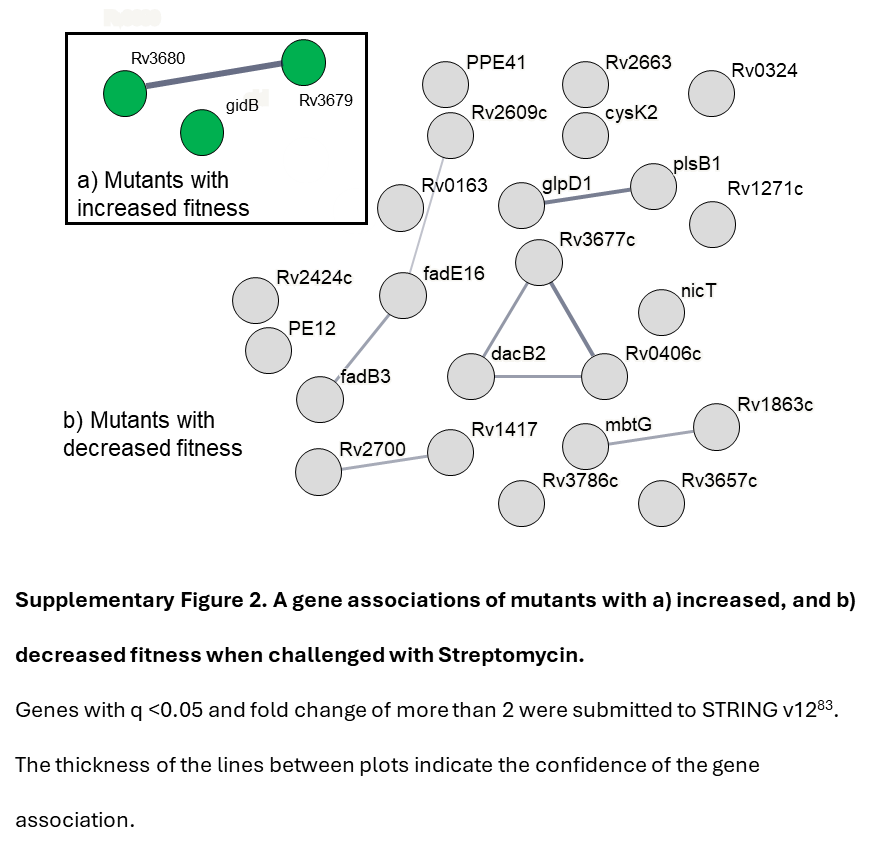


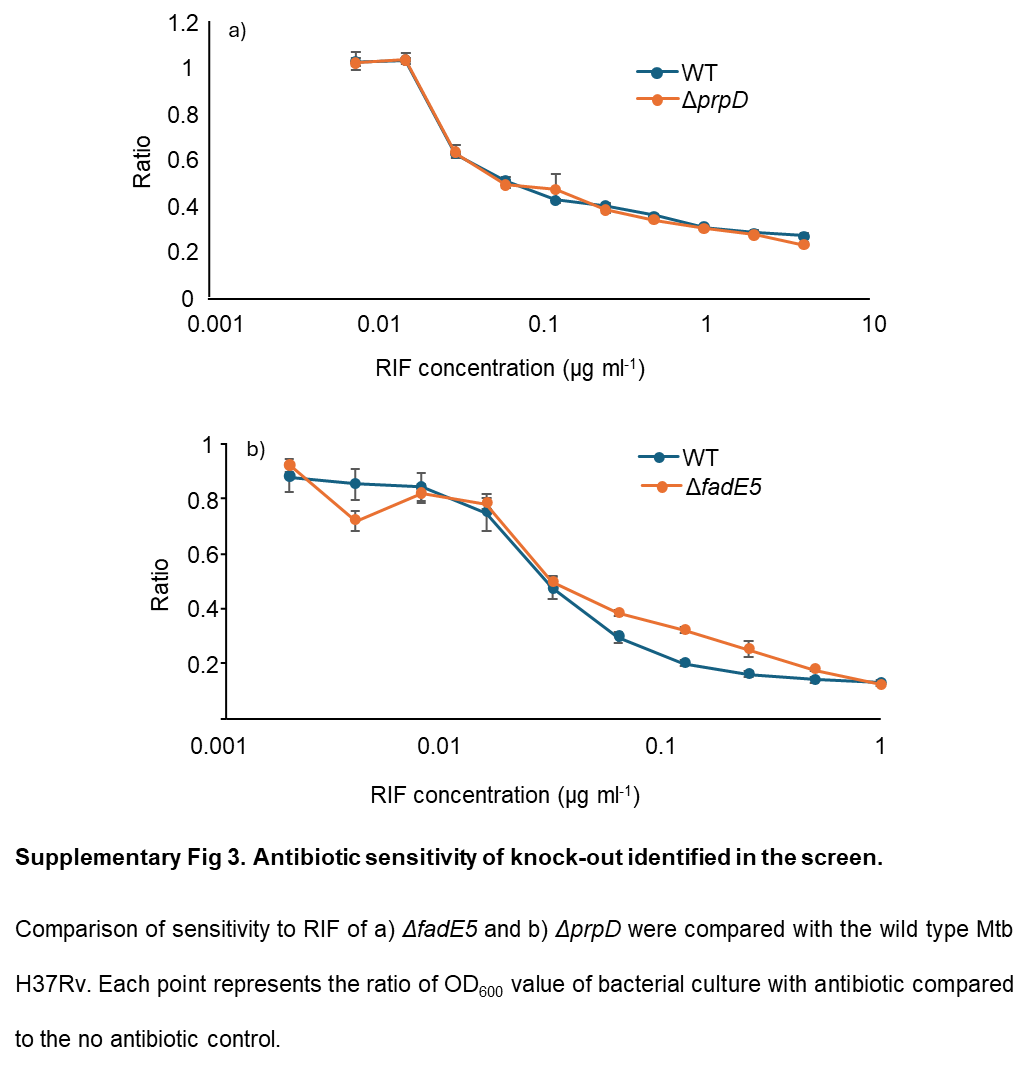


**Supplementary Table 1: Primers for Tn-seq analysis**

Primers Mar A – Mar J contain the **Illumina binding region,** an index to allow multiplexing, the Illumina read sequence, a **spacer** to allow the sequencer to keep track of sequencing spots and the transposon specific sequence. PHO (bold text corresponds to the 5’ Illumina binding region, the NNNNNNN index allows removal of PCR amplification artefacts, therefore products with an identical position and index to an already identified product are discarded, and the red the Illumina read sequence. Lower case indicates the phosphorothioates (PTO) bonds to prevent any endonuclease degradation). The PHO is 3’ phosphate to prevent extension of the adapter by polymerases.

| MarA | **AATGATACGGCGACCACCGAGATCTACACTGTTCCGA**ACACTCTTTCCCTACACGACGCTCTTCCGATCT**CGGGGACTTATCAGCCAACC** |
| --- | --- |
| MarB | **AATGATACGGCGACCACCGAGATCTACACTTCCGGAG**ACACTCTTTCCCTACACGACGCTCTTCCGATCT**TCGGGGACTTATCAGCCAACC** |
| MarC | **AATGATACGGCGACCACCGAGATCTACACGCCGATGT**ACACTCTTTCCCTACACGACGCTCTTCCGATCT**GATACGGGGACTTATCAGCCAACC** |
| MarD | **AATGATACGGCGACCACCGAGATCTACACCATGATCG**ACACTCTTTCCCTACACGACGCTCTTCCGATCT**TATCTACGGGGACTTATCAGCCAACC** |
| MarE | **AATGATACGGCGACCACCGAGATCTACACCGCGCGGT**ACACTCTTTCCCTACACGACGCTCTTCCGATCT**CGGGGACTTATCAGCCAACC** |
| MarF | **AATGATACGGCGACCACCGAGATCTACACACACGATC**ACACTCTTTCCCTACACGACGCTCTTCCGATCT**TCGGGGACTTATCAGCCAACC** |
| MarG | **AATGATACGGCGACCACCGAGATCTACACAAGTAGAG**ACACTCTTTCCCTACACGACGCTCTTCCGATCT**GATACGGGGACTTATCAGCCAACC** |
| MarH | **AATGATACGGCGACCACCGAGATCTACACGAGATCTT**ACACTCTTTCCCTACACGACGCTCTTCCGATCT**TATCT**A**CGGGGACTTATCAGCCAACC** |
| MarJ | **AATGATACGGCGACCACCGAGATCTACACAGATCGCA**ACACTCTTTCCCTACACGACGCTCTTCCGATCT**TATCT**A**CGGGGACTTATCAGCCAACC** |
| IS6 | CAAGCAGAAGACGGCATACGA |
| Adap1 | caagc**AGAAGACGGCATACGAGAT**NNNNNNNNGTGACTGGAGTTCAGACGTGTGCTCTTCCgatct |
| Adap2 | gatcgGAAgagca |

**Supplementary Table 2: Primers for *prpD* replacement knockout**

Primers used to amplify homologous regions upstream and downstream of *prpD*(Rv1130). Restriction enzyme recognition sites are in bold

| Rv1130UpF (AflII) | gcatga**cttaag**agtagttggccagtccgatg |
| --- | --- |
| Rv1130UpRev (XbaI) | gcatga**tctaga**caaattttgcgaacatgacg |
| Rv1130DownFw (HindIII) | gcatga**aagctt**gcagaccaattcgttgacct |
| Rv1130DownRev (SpeI) | gcatga**actagt**aaatcgaggttgggcaagat |
